# 

**Published:** 1906-09-08

**Authors:** 


### BOOKS RECEIVED.

Henry Kimpton, London.

"Food and Digestion in Health and Disease." By M. A. Dutch, M.D.

"Practical Text-book of Midwifery for Nurses." By Robert Jardine, M.D.Edin. Price 5s. net.

John Wright and Co., Bristol.

"Indications for Operations in Diseases of the Internal Organs." By Professor Hermann Schlesinger, M.D. English edition by Keith N. Monsarrat, M.B., F.R.C.S., Edin. Price 9s. 6d. each net.

Baillière, Tindall, and Cox.

"Anæsthetics." By J. Blumfeld, M.D. Second edition.

J. and A. Churchill.

"The Use of Shower Baths in Schools in England and on the Continent." By F. Rose, Ph.D.

The Scientific Press, Ltd.

"Nursing. Its Principles and Practice." By Isabel Hampton Robb. Third Edition. Revised and enlarged.

F. A. Davis and Co., Philadelphia.

"Christianity and Sex Problems." By H. Northcote, M.A.

### DEATH.

**Notices of Births, Deaths, and Marriages** are inserted in this column at a charge of 2s. 6d. for an announcement not exceeding four lines.

On the 21st ult., at 65 Frogmore Street, Abergavenny, Elleanor Norton, late Sister at Manchester Royal Eye Hospital. (2554)

### NOTICE TO CORRESPONDENTS.

All MSS., Letters, Books for Review, and other matters intended for the Editor should be addressed The Editor, "The Hospital," The Hospital Buildings, 28 & 29 Southampton Street, Strand, W.C.

The Editor cannot undertake to return rejected MS., even when accompanied by stamped directed envelope.

**Notice to Advertisers and Subscribers.**

All Advertisements, Orders for Copies of The Hospital, and Business Communications should be addressed to **THE MANAGER (Not to the Editor),** The Hospital, 28 & 29 Southampton Street, Strand London, W.C.

The Cost of Subscription, post free, is as follows:—Per week, 3½d.; per quarter, 3s. 3d.; per half-year, 6s. 6d.; per annum, 13s. Foreign and Colonial:—Per annum, 19s. 6d., payable, in all cases, in advance.

**NOTICE.—Vol. XXXIX. of "The Hospital" (October 1905 to March 1906), in handsome cloth binding, will shortly be on sale at the office of this Journal, price 10s. 6d. Cases for binding the Numbers contained in this Volume 2s. Index to Vol. XXXIX., 3½d., post free.**

**The Monthly Part for September is now ready. Price 1s., by post, 1s. 3d.**

### CHARTS

### MORNING & EVENING

Arranged for four weeks.

### FOUR-HOUR CHARTS

For recording Temperatures at the hours of

**2, 6, 10, or 12, 4, 8,**

for a period of nine complete days (i.e., day and night).

Price, Carriage paid:—

| | |
|---|---|
| 25 ... ... ... | **1/0** |
| 50 ... ... ... | **1/6** |
| 100 ... ... ... | **2/9** |
| 250 ... ... ... | **6/6** |
| 500 ... ... ... | **12/0** |
| 1,000 ... ... ... | **23/0** |

These Charts are especially prepared for Institutions and Private Nurses to a standard size of 10 in. by 8 in. They are clearly printed on good paper, and will be found to be the cheapest and most serviceable Charts published.

**JAPANNED TIN WASHABLE CHART-HOLDERS.**

Specially designed for the above Charts. Stoutly made, easily cleaned or sterilized.

**1/-** each, **9/6** per dozen, carriage paid.

**A Useful Novelty for District, Private, and Maternity Nurses.**

**THE POCKET-BOOK OF TEMPERATURE CHARTS.**

Containing 17 Twelve-hour and 8 Four-hour Charts, perforated so that each Chart may be removed after use. Size 8 by 5. Can be easily folded and carried in the cloak or apron pocket.

**6d.** net, **7d.** post free.

### CASE-BOOKS

**A CASE-BOOK FOR PRIVATE NURSING.**

Arranged for Day and Night Nurse's Report, with rulings in accordance with what has been found in practice to be the most suitable and approved system for Pay Hospitals, Nursing Homes, Private Nurses, &c. The size of an Exercise Book, 7½ in. by 9½ in., containing 48 pp.

**This Case-book has met with the highest praise from foremost Members of the Medical Profession.**

**3d.** net, **4½d.** post free. Special quotations for a quantity.

**THE POCKET CASE-BOOK FOR DISTRICT AND PRIVATE NURSES.**

By a PHYSICIAN. Suitable size for apron pocket.

Cloth boards, **1/-** net; in handsome leather, gilt, **1/6** net, **1/7** post free.

**THE "SIMPLEX" DISTRICT NURSES' CASE-BOOK.**

Containing space for 50 Cases, ruled and printed. Strongly bound in cloth boards. Size, 6 by 4.

**6d.** net, **7d.** post free.

*Complete Catalogue of Nursing Manuals, &c., post free on application.*

**The Scientific Press, Ltd.,**

**Publishers of Nursing Text-Books, Charts, and Case-Books of all Descriptions.**

**28 & 29 SOUTHAMPTON STREET, STRAND, LONDON, W.C.**

"An invaluable handbook for all engaged in philanthropic work."
ST. JAMES'S GAZETTE.

### BURDETT'S HOSPITALS & CHARITIES, 1906.

BEING

The Year Book of Philanthropy and the Hospital Annual.

Over 1,000 pages.
Price 6/- net; 6/5 post free.

*Of all Booksellers, or of*

THE SCIENTIFIC PRESS, Ltd.,
28 and 29 Southampton Street, Strand, London, W.C.

